# 776 Quality Improvement in Prehospital Burn Care

**DOI:** 10.1093/jbcr/irac012.329

**Published:** 2022-03-23

**Authors:** Frank Phillips, Mariah Sturges

**Affiliations:** Allegheny Health Network, Pittsburgh, Pennsylvania; AHN West Penn, Pittsburgh, Pennsylvania

## Abstract

**Introduction:**

Burn center implemented a new quality improvement program with emergency medical services (EMS) that examined patient care prior to arrival. Inconsistencies were noted with key interventions that would improve patient’s overall health. These included oxygen administration, fluid resuscitation, pain management, and the application of sterile dressings. There was also improvement in the transfer of care from EMS to receiving hospital.

**Methods:**

Patient care reports were examined to see the quality of care that was being given in the prehospital setting. Based on these results, education in the form of a lecture and prior cases are presented routinely to different emergency medical services. To improve transfer of care at bed side from EMS, “burn page” was created. This is a page sent directly to charge nurse of burn unit, emergency department, attending physicians of burn unit, outreach coordinator, and prehospital care coordinator. This allows for improved preparation to receive burn patient from EMS. After the patient is received from EMS, a patient follow up is provided by the prehospital care coordinator to EMS within 24 hours. The patient care report from EMS is reviewed along with the care currently being provided by the burn unit. A case review is scheduled with EMS to examine the care provided and see where improvement can be made with both EMS care and the transition of care at the bed side. This case review also allows the EMS crew to see how the patient is doing a few weeks later.

**Results:**

The burn center has noticed drastic improvement of care in the prehospital setting. This includes hitting critical bench marks as described in the introduction. This improvement is seen at the transition of care at bedside and in patient care reports done by EMS. These EMS providers also express the improvement of how patients are transferred to the burn center and a sense of trust that the patient will have excellent burn care. They also express that creating the patient follow ups and case reviews helps close the loop in patient care resulting in better burn care being provided in the prehospital setting.

**Conclusions:**

With noticing what critical bench marks that were not being met regarding burn care in the prehospital setting, corrective actions can be created that will establish a closed loop that drastically improves care. From the point of injury to the end of stay in the burn unit, patient care has been drastically improved.

